# T Cells Contribute to Tumor Progression by Favoring Pro-Tumoral Properties of Intra-Tumoral Myeloid Cells in a Mouse Model for Spontaneous Melanoma

**DOI:** 10.1371/journal.pone.0020235

**Published:** 2011-05-25

**Authors:** Renée Lengagne, Arnaud Pommier, Jonathan Caron, Laetitia Douguet, Marylène Garcette, Masashi Kato, Marie-Françoise Avril, Jean-Pierre Abastado, Nadège Bercovici, Bruno Lucas, Armelle Prévost-Blondel

**Affiliations:** 1 INSERM, U1016, Institut Cochin, Paris, France; 2 CNRS, UMR8104, Paris, France; 3 University Paris Descartes, Paris, France; 4 Unit of Environmental Health Sciences, Chubu University, Aichi, Japan; 5 APHP, Hôpital Cochin, Service de Dermatologie, Paris, France; 6 Singapore Immunology Network, BMSI, A-STAR, Singapore, Singapore; Karolinska Institutet, Sweden

## Abstract

Tumors affect myelopoeisis and induce the expansion of myeloid cells with immunosuppressive activity. In the MT/ret model of spontaneous metastatic melanoma, myeloid cells are the most abundant tumor infiltrating hematopoietic population and their proportion is highest in the most aggressive cutaneous metastasis. Our data suggest that the tumor microenvironment favors polarization of myeloid cells into type 2 cells characterized by F4/80 expression, a weak capacity to secrete IL-12 and a high production of arginase. Myeloid cells from tumor and spleen of MT/ret mice inhibit T cell proliferation and IFNγ secretion. Interestingly, T cells play a role in type 2 polarization of myeloid cells. Indeed, intra-tumoral myeloid cells from MT/ret mice lacking T cells are not only less suppressive towards T cells than corresponding cells from wild-type MT/ret mice, but they also inhibit more efficiently melanoma cell proliferation. Thus, our data support the existence of a vicious circle, in which T cells may favor cancer development by establishing an environment that is likely to skew myeloid cell immunity toward a tumor promoting response that, in turn, suppresses immune effector cell functions.

## Introduction

Tumor development affects bone marrow myelopoeisis and induces the expansion of myeloid derived suppressive cells (MDSC) [1]. In mouse models, MDSC express the αM integrin CD11b and the myeloid lineage differentiation antigen Ly6C/G recognized by the Gr1 antibody. CD11b^+^Gr1^+^ cells represent 2–4% of splenocytes of normal mice, but increase 5- to 20- fold in mice transplanted with tumors [2]. Tumor-induced CD11b^+^Gr1^+^ splenocytes are considered to be a heterogeneous population of immature monocytes/macrophages, granulocytes, dendritic cells and their progenitors [3], [4], [5]. It is established that MDSC suppress conventional T cell proliferation and activation [6]. In addition, MDSC have been suggested to impact the functions of other cells including NK, macrophages and regulatory T cells [7], [8], [9]. Dissection of their properties is hampered by the lack of specific marker. The α chain of the IL4 receptor (IL-4Rα) [10] and the M-CSF receptor (CD115) [11] have been proposed to help identifying subpopulations of mouse MDSC. Nevertheless, they do not identify immune suppressive populations of MDSC in various tumor models [12].

Macrophages have long been recognized as important components of the innate immunity against tumors [13]. While tumor associated macrophages (TAM) can prevent the establishment and spread of tumor cells, they may also favor tumor growth and dissemination. This paradox is due to the inherent plasticity of macrophages, which can display distinct phenotypes and functions in response to different signals [14], [15], [16]. M1 macrophages secrete IL-12 and NO, promote antitumor immunity and directly kill tumor cells, whereas alternatively activated M2 macrophages exhibit defective production of IL-12, high IL-10 secretion, produce arginase, suppress the antitumor response and promote angiogenesis and metastasis [17], [18], [19], [20]. Accordingly, TAM favor tumor progression in most mouse models [21], [22], [23], [24], but are vital for tumor eradication in others [25], [26], suggesting that TAM display contradictory roles depending on the tumor type which might dictate their polarization [27].

Only few recent data have shown that immune cells within the tumor microenvironment may influence the pro-tumoral properties of tumor infiltrating macrophages. De Visser et al were the first to demonstrate the requirement of B cells in mediating the recruitment of inflammatory cells into premalignant skin associated with neoplastic progression using the model of HPV16 induced epithelial carcinogenesis [28]. More recently, B cells have been shown to play a role in driving M2 like polarization of TAM [29]. Sinha et al have shown that MDSC suppress tumor immunity by reducing macrophage IL12 production via an IL10 dependent mechanism [9]. In addition, although some subsets of lymphocytes including cytotoxic CD8^+^ T cells and NK cells exhibit anti-tumor immunity [30], other subsets notably Th2 cells, CD4^+^ regulatory T cells and NKT cells may exhibit opposite effect on tumor progression by interfering with TAM properties [31]. Thus, various immune cells seem to contribute to myeloid orientation although the relative contribution of the different cell types during spontaneous development of tumors is unclear.

In the model of spontaneous melanoma driven by the RET oncogene (MT/ret mice; [32]), the primary uveal tumor cells disseminate at three weeks of age, but remain dormant for several weeks [33]. 50% of 3 month old mice display cutaneous metastasis and finally develop visceral metastasis [34]. In this MT/ret model, we have shown that CD8^+^ T cell depletion does not accelerate the onset of cutaneous metastasis, suggesting the presence of tumor induced immunosuppressive factors locally. In the present study, we focused our interest on the characterization of myeloid cells within cutaneous metastasis. Given the critical role of adaptative immunity in regulating innate immune cell functions in some mouse models of cancer development [14], we addressed the possibility that T cells might exert a role in regulating recruitment and/or pro-tumor properties of tumor infiltrating myeloid cells in MT/ret mice. We report that myeloid cells are the most abundant hematopoietic population within the cutaneous metastasis and that they display immune suppressive functions. Our data further established the critical role of T cells in the acquisition of pro-tumoral properties of intra-tumoral myeloid cells in the course of melanoma development.

## Materials and Methods

### Ethics statement

All animals were handled in strict accordance with good animal practice in compliance with French Ministry of Agriculture regulations for animal experimentation. The animal experiment protocol approval number is 75–510 and was delivered by the veterinary departement of Paris. All experiments were performed in animal facilities which also received an approval number (A75-14-02).

### Mice

MT/ret transgenic mice express the human Ret oncogene [32]. 3 to six month old mice at different stages of malignancy were used and age-matched non-transgenic MT/ret^−/−^ littermates were used as control (ctrl). MT/ret mice were crossed with C57BL/6 CD3ε^−/−^ mice [35] to obtain RetCD3εKO and RetCD3ε^+/−^ (called RetCD3ε^+^ later) mice. MT/ret, RetCD3εKO and RetCD3ε^+^ mice were diagnosed for recording the development of exophthalmus related to the uveal primary melanoma, and subsequent cutaneous metastasis. All mice are maintained in our own pathogen free animal facilities. OT-1 mice expressing a TCR specific for Ova257-264 were purchased from Charles River Laboratories.

### Cell suspension procedures from lymphoid organs and tumors

Spleens and cutaneous tumor masses were mechanically dissociated and digested with 1mg/mL collagenase A and 0,1mg/mL DNase I (Roche, Mannheim, Germany) for 25 min at 37°C. Single cell suspensions were filtered, washed in PBS 1X, 5% FCS, 0.5 mM EDTA and resuspended in RPMI 1640.

### Flow cytometry

After blocking with anti-FcγR Ab, cell suspensions were stained with the following mAbs against CD45.2, CD11b, Gr1, TcRαβ, TcRγδ, CD8α, CD4, CD19 and CD124 from Pharmingen (BD Biosciences, Le Pont de Claix, France), NK1.1 from eBiosciences (San Diego, CA) and F4/80 from Serotec (Düsseldorf, Germany). For IL-12 intracytoplasmic staining, cell suspensions were stimulated overnight with 2 µM Golgi Stop Monensin, 0.1 µg/ml LPS and 101ng/ml IFNγ. The staining was performed following the manufacturer’s instructions (kit Cytofix/cytoperm, BD Biosciences) using the combination CD45.2, CD11b, and IL-12. Analyses were performed on a FacsCalibur cytofluorometer (BD Biosciences).

### Isolation of myeloid cells and supernatants

CD11b^+^ cells from spleens and cutaneous nodules were selected via magnetic microbeads conjugated to anti-mouse CD11b mAb (Mac-1; Miltenyi Biotec) using MS columns according to the manufacturer’s specifications (Miltenyi Biotec). For supernatant collection, 5.10^5^ cells per ml were cultured for 48 h in complete medium at 37°C.

### IFNγ-ELISpot

Ctrl mice were immunized with 50 µg of peptide 33–41 from LCMV glycoprotein (GP33:KAVYNFATM) in IFA. Eight days later, inguinal lymph node cells were collected. ELISpot was performed using the mouse IFNγ ELISpot kit (BD Biosciences). GP33-specific T cells (2.10^5^ cells/well) were stimulated 24 h with GP33 (10-7M) in the presence of CD11b^+^ or CD11b^-^ cells (10^4^ cells/well) or corresponding myeloid derived supernatants. In another setting, freshly isolated splenocytes (2.10^5^ cells/well) from tumor bearing mice were cultured with the syngeneic TIII melanoma cell line (10^3^ cells/well) [34]. Splenocytes were used either directly or after depletion of CD11b^+^ cells.

### CFSE labeling and IFNγ secretion of TCR transgenic T cells

CD8 T cells from lymph nodes of OT-1 mice were prepared using a mouse CD8 negative isolation kit (Dynal Biotec, Oslo, Norway). Purified cells were labeled with CFSE (Molecular Probes). 5.10^4^ CFSE^+^ cells were cultured for 72 h in presence or not of Ova257 (8.10^−4^ µM) with 5.10^5^ CD11b^+^ cells. The supernatants were analyzed for IFNγ by ELISA using IFNγ capture biotinylated mAb, and streptavidin-alkaline phosphatase (BD biosciences). CFSE dilution was determined by flow cytometry.

### Quantitative RT-PCR

Total RNA was isolated from purified CD11b^+^ cells using RNAlater and RNeasy columns (Qiagen, Courtaboeuf, France). RNA was reverse transcribed with SuperScript™ II (Invitrogen) and oligo-dT18 primers. Quantitative PCR was carried out using fast SYBR Green Master Mix (Applied Biosystems) and a real time PCR system (Light Cycler 1.5, Roche Diagnostics, Division Applied Sciences, Meylan, France) according to standard PCR conditions. For quantitative calculations, values were normalized to GAPDH expression. Primer sequences are listed in Table 1.

**Table 1:** Primer pairs used for real time PCR experiments.

Target cDNA	Upper/Lower	Sequences (5’ to 3’)
GAPDH	U	GCC GGT GCT GAG TAT GTC GT
	L	GGA GAT GAT GAC CCG TTT GG
IL10	U	GGT TGC CAA GCC TTA TCG GA
	L	ACC TGC TCC ACT GCC TTG CT
ARG1	U	ATG GAA GAG ACC TTC AGC TAC
	L	GCT GTC TTC CCA AGA GTT GGG
FIZZ1	U	CCC AGG ATG CCA ACT TTG AA
	L	GGC CCA TCT GTT CAT AGT CT
MGL1	U	ATG ATG TCT GCC AGA GAA CC
	L	ATG ATG TCT GCC AGA GAA CC
EMR1	U	CTC ACC GGT ATA GAC AA
	L	GCA GGC GAG GAA
CCL2	U	TTA AAA ACC TGG ATC GGA ACC AA
	L	GCA TTA GCT TCA GAT TTA CGG GT

### Tumor cell proliferation assay

The xCELLigence System (Roche Diagnostics) monitors cellular events in real time without the incorporation of labels. The System measures electrical impedance across interdigitated micro-electrodes integrated on the bottom of tissue culture E-Plates. The impedance measurement provides quantitative information about the status of the adherent cells, including cell number, viability, and morphology. Melan-ret melanoma cells (5.10^3^) were seeded into the wells of 96X E-Plates in 100 µl of media. Cell adhesion and growth were monitored 48h till their exponential growth phase. Tumor derived CD11b^+^ purified cells (5.10^4^ cells) were added in a volume of 100 µl/well. Co-cultures were assessed by the system with a measure every 5 min for up to 40 h. Results, expressed as Cellular Index, were normalized (nCI) with RTCA Software, and expressed as percentage of specific inhibition  =  (1-nCI (Melan-ret cells + CD11b^+^ cells) /nCi (Melan-ret cells))×100.

### NO assay

Purified CD11b^+^ cells (5.10^5^) were cultured for 2 days in RPMI, 10% FCS supplemented with LPS and IFNγ. NO was measured using Griess reagents (Promega, Charbonnières-les-Bains). Briefly, 50 µl of culture supernatant was incubated for 10 min at room temperature with 50 µl of Griess reagent A plus 50 µl of Griess reagent B. Absorbance at 540 nm was measured using a microplate reader (Perkin Elmer). Data are mean +/−SD of triplicate wells.

### Statistical analysis

Statistical analysis was performed using the GraphPad Prism 4.0 software (San Diego, CA).

## Results

### CD11b^+^Gr1^high^ myeloid cells accumulate in the spleen in the course of natural melanoma progression

**Figure 1:**
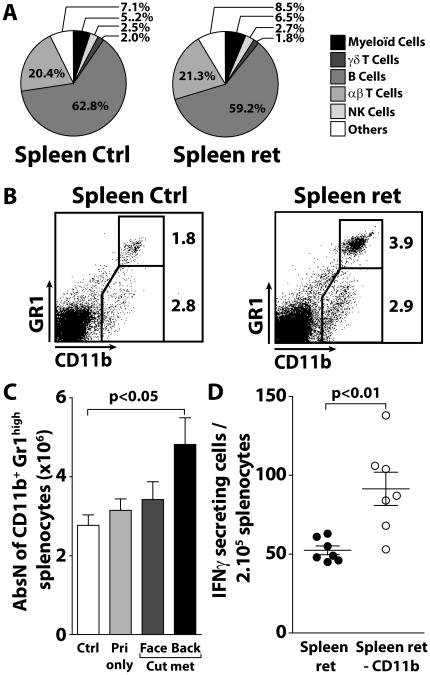
Accumulation of CD11b^**+**^Gr1^high^ myeloid cells in spleen of tumor bearing MT/ret mice. (**A**) Comparison of the proportion of hematopoietic cells in spleen of MT/ret and ctrl mice. Myeloid cells are CD11b^+^ cells. αβT cells are defined as CD4^+^ αβTCR^high^ cells and CD8^+^ αβTCR^high^ cells. γδT cells are defined as CD8- γδTCRhigh cells. B cells are defined as CD19^+^CD8^-^ cells and NK cells are NK1.1^+^CD4^-^CD8^-^ cells. The pie diagram summarizes data from spleens of MT/ret (n = 29) and Ctrl (n = 13) age matched mice. (**B**) Single cell suspensions derived from spleens of MT/ret or Ctrl mice were stained for CD45, CD11b and Gr1. Representative CD11b/Gr1 dot plots were generated from gated CD45^+^ cells. (**C**) The histograms correspond to the absolute numbers of both CD11b^+^Gr1^high^ cell subsets in ctrl spleens (n = 13) and in spleens of MT/ret mice displaying either primary tumors (n = 8), facial (face, n = 13) or dorsal cutaneous metastasis (back, n = 12). (**D**) Myeloid cells from MT/ret spleen inhibit the spontaneous response specific for melanoma. The number of IFNγ-secreting cells was assessed by an ELISPOT assay using splenocytes from MT/ret mice as effectors and Melan-ret cells as targets. Ex vivo splenocytes were used either in total or after depletion of CD11b^+^ cells. The significance was assessed using unpaired t test.

The MT/ret model allowed the monitoring of immune cells within the spleen and the tumor microenvironment during the course of spontaneous tumor progression. Exophthalmus corresponds to the first clinical sign of uveal primary melanoma development. Within 3 months after birth, 50% of mice display cutaneous metastasis that develop first on the face, then in the posterior part of the body [34]. The proportions of hematopoietic populations within spleens derived from age matched MT/ret and ctrl mice were not statistically different (Fig. 1A). However, CD11b^+^ cells consist of two main populations according to Gr1 expression level, Gr1^low^ (monocytic) and Gr1^high^ (granulocytic), and the proportions of CD11b^+^ subsets in MT/ret mice differ from those in ctrl mice. More precisely, CD11b^+^Gr1^high^ cells accumulate in spleen of mice displaying dorsal metastasis corresponding to a late melanoma stage (Fig. 1B and C). In addition, we have previously shown that MT/ret mice develop anti-tumor immune response spontaneously during disease progression [34]. To evaluate if this anti-tumor immune response is negatively influenced by myeloid cells in the spleen, either total splenocytes or CD11b^+^ cell-depleted splenocytes were stimulated with Melan-ret melanoma cells. The removal of CD11b^+^ cells raises the number of splenocytes responding to melanoma cells (Fig. 1D). Together our data indicate that, as shown in models of tumor transplantation, myeloid cells accumulate within spleen of MT/ret mice and prevent optimal anti-tumor T cell response.

### Accumulation of myeloid cells within cutaneous metastasis correlates with the tumor aggressiveness

**Figure 2:**
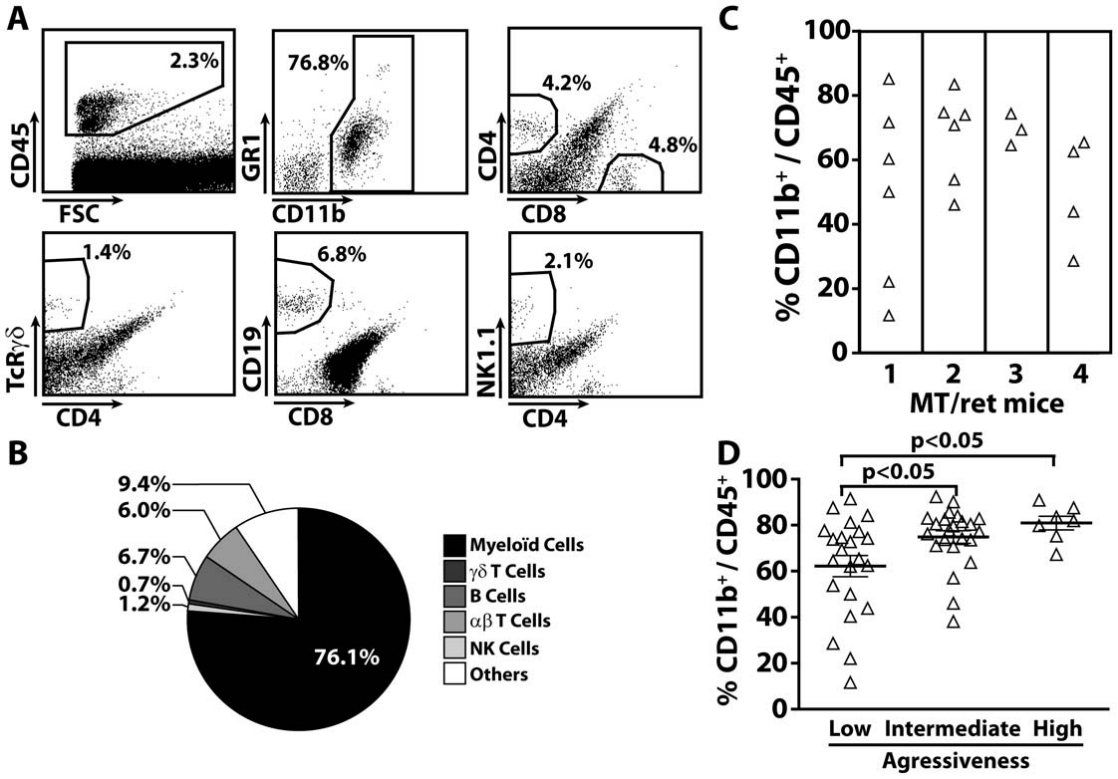
Characterization of hematopoietic cells within the tumor microenvironment of MT/ret mice. (**A**) Dot plots from a cell suspension of one representative cutaneous metastasis. Myeloid and lymphoid stainings were performed as defined in Fig 1A. (**B**) The pie diagram summarizes the proportion of hematopoietic cells from 47 cutaneous metastasis. (**C**) Variability of the proportion of tumor infiltrating CD11b^+^ cells from cutaneous metastasis. The frequencies of CD11b^+^ cells gated from CD45^+^ cells were determined. The graph shows the data for four 3 month old mice. (**D**) Correlation between the tumor aggressiveness and the increase of tumor infiltrating CD11b^+^ cells. The tumor aggressiveness corresponds to the ratio between the absolute numbers of tumor associated cells and the number of days since appearance of each nodule. Statistical differences were assessed using ANOVA test.

To extent these data to the monitoring of the tumor microenvironment, we first compared the proportion of hematopoietic cells that infiltrate cutaneous metastasis derived from 3 to 6 month old MT/ret mice (Fig. 2*A, 2B*). CD45^+^ cells represent 2.3% of total cells. αβT and B cells represent on an 6+/−0.5% and 6.7+/−0.8% of hematopoietic cells respectively. The percentages of γδT and NK cells are pretty low. More interestingly, the percentage of CD11b^+^ cells ranges from 11.6 to 92.4% with an average 76.1% of CD45^+^ cells and they are almost exclusively Gr1^low^. In a given mouse, the proportion of CD11b^+^ cells could be variable from tumor to tumor as shown in Fig. 2*C* for 4 mice. In order to evaluate the association of tumor infiltrating myeloid cells with tumor progression for one given nodule, we defined a “tumor aggressiveness score” corresponding to the ratio between the absolute number of cells in the tumor and the number of days since its appearance. The most aggressive tumors (>2.10^5^ cells/day) displayed a high proportion of CD11b^+^ cells, whereas the less aggressive ones (<2.10^4^ cells/day) are significantly less infiltrated by myeloid cells (Fig. 2*D*). Tumors with intermediate aggressiveness already displayed an increased proportion of CD11b^+^ cells.

### Tumor infiltrating myeloid cells express markers of M2 type macrophages

**Figure 3:**
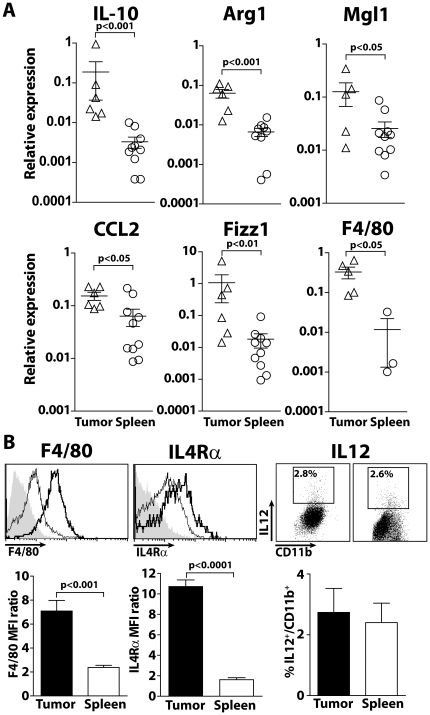
Characterization of myeloid cells from MT/ret mice. (**A**) QPCR. CD11b^+^ cells were isolated from tumors and spleens of tumor bearing MT/ret mice. The transcripts levels of a panel of genes were analyzed by RT-PCR. Mean values+/− SEM of relative expression are shown for indicated genes. (**B**) Phenotype and function of CD11b^+^ cells. Cell suspensions from tumors and spleens of MT/ret mice were stained for CD45, CD11b and IL4-Rα, F4/80 and IL12 and their isotype controls (grey histogram). Representative stainings for spleen (single line) and tumor (bold line) are shown. IL-12/CD11b^+^ dot plots generated from gated CD45^+^ cells are obtained after stimulation with IFNγ and LPS. Representative histograms of more than 3 experiments and performed on more than 10 samples are shown. Results are expressed as the percentage of IL-12^+^ cells from CD45^+^CD11b^+^Gr1^+^ cells taking account the two Gr1 subsets within spleens. Statistical differences were assessed using unpaired t test.

To further compare myeloid cells that accumulate during tumor progression, quantitative PCR were carried out on CD11b^+^ purified cells from spleen and tumor samples using a set of type 2 myeloid-associated marker genes. QPCR analysis revealed that il10, arginase I, mgl1 fizz1 and the inflammatory chemokine ccl2 mRNA levels were all significantly higher in tumor derived CD11b^+^ cells (Fig. 3*A*). In addition, these cells were strongly positive for F4/80 mRNA compared to related cells in spleen. Flow cytometric analysis further showed that tumor infiltrating myeloid cells express F4/80 at the protein level, revealing a significant upregulation of this macrophage marker in the tumor microenvironment (Fig. 3*B*). In addition, tumor infiltrating myeloid cells express IL-4Rα (Fig. 3B). Contrasting with transplanted tumor models [12], IL-4Rα expression in spleen of tumor-bearing MT/ret mice is low (Fig. 3B) and similar to the level observed in splenocytes from control mice (data not shown). A relatively low proportion of tumor infiltrating myeloid cells secrete IL-12 upon a short IFNγ/LPS stimulation (2.7+/−0.8; Fig. 3B), *a pro*portion quite similar to that of related splenic myeloid cells. Overall, tumor infiltrating myeloid cells are enriched in F4/80^+^, IL-4Rα^+^ cells and only a minority of them have the capacity to produce IL-12.

### Tumor and spleen derived myeloid cells impairs T cell functions

**Figure 4:**
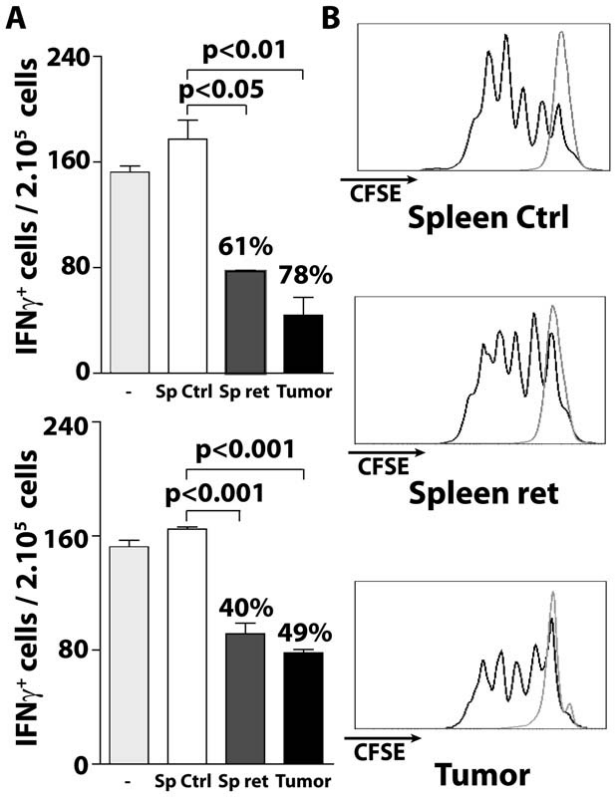
CD11b^**+**^ cells from MT/ret mice suppress T cell functions. (**A**) GP33-specific T cells from GP33-immunized mice were stimulated 24 h with GP33 in presence of CD11b^+^ cells isolated either from tumors or spleens of MT/ret mice or ctrl spleens. The frequency of IFNγ secreting T cells was determined by an ELISPOT assay. The percentage of inhibition indicated on the graph corresponds to the ratio between the number of spots in presence and in absence of CD11b^+^ (upper histogram). GP33-specific T cells were also stimulated with GP33 together with supernatants of CD11b^+^ cells isolated from tumors or spleens of MT/ret or non transgenic mice and tested as above (lower histogram). (**B**) Purified OT-1 CD8^+^ T cells labeled with CFSE were cultured in presence of CD11b^+^ cells isolated from spleens or tumor nodules from MT/ret mice or from ctrl spleen, and stimulated in presence or not of Ova257. Three days later, proliferation was determined. CFSE fluorescences are shown after culture with (bold lines) or without Ova257 (thin lines).

To compare the impact of myeloid cells from tumor bearing MT/ret mice on T cell functions, we first stimulated T cells from GP33 immunized mice with GP33 in the presence of CD11b^+^ cells. CD11b^+^ cells isolated from tumors or spleens of MT/ret mice inhibit IFNγ secretion (78% and 61% inhibition respectively) (Fig. 4*A* *, upper histogram*). Supernatants from tumor- or spleen-derived CD11b^+^ cells of MT/ret mice also reduced the proportions of IFNγ secreting T cells (49% and 40% inhibition respectively), while supernatant from control mice had no effect (Fig. 4*A* *, lower histogram*). In addition, we cultured CD11b^+^ cells with CD8^+^ T cells specific for Ova257 peptide from OT-1 mice. In the presence of Ova257 and control CD11b^+^ cells, a majority of OT-1 cells undergoes three to four cycles, whereas CD11b^+^ cells derived from tumors or spleens of MT/ret mice reduced Ova257 specific T cell division (Fig. 4*B*). Together our data indicate that despite their phenotypic differences described above, both splenic and tumor derived myeloid populations inhibit CD8^+^ T cell proliferation and IFNγ secretion.

### T cells contribute to the immunosuppressive profile of tumor infiltrating myeloid cells

**Figure 5:**
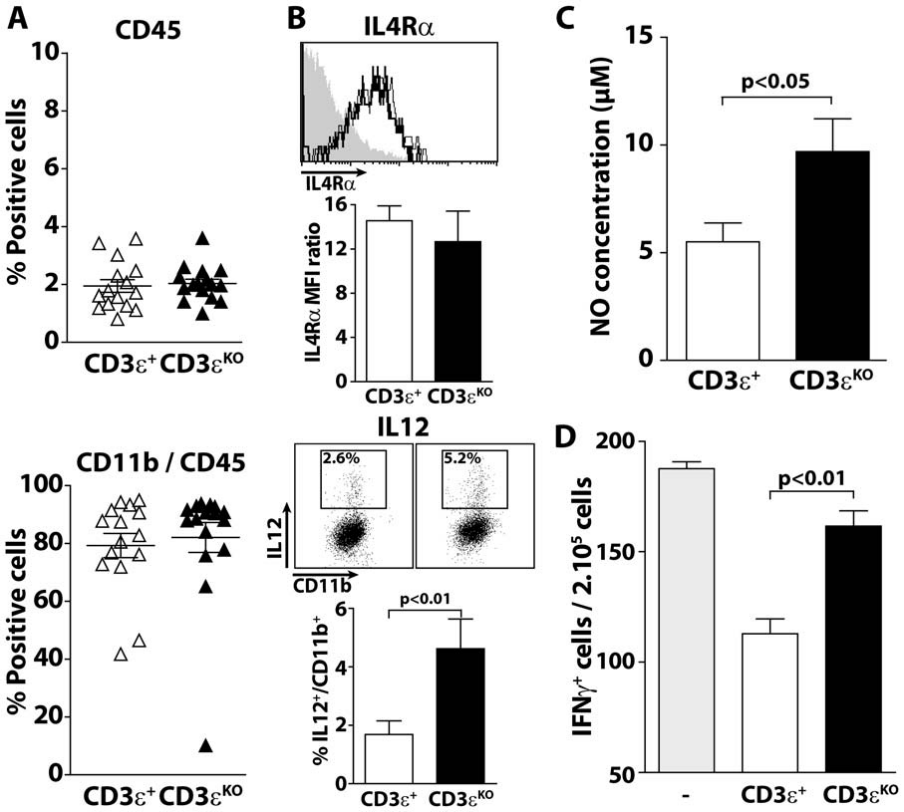
T cells contribute to the immunosuppressive function of tumor infiltrating myeloid cells. (**A**) The graphs indicate the proportion of CD45^+^ cells from live cells and myeloid cells from CD45^+^ cells from tumors of RetCD3ε^+^ (n = 15) and RetCD3εKO (n = 16) age matched mice. (**B**) Cell suspensions from tumors derived from RetCD3ε^+^ and RetCD3εKO mice were stained for CD45, CD11b and IL4-Rα. Representative histograms are shown for IL4-Rα expression from CD45^+^CD11b^+^ cells. The histograms below summarize the MFI ratio of IL4-Rα specific staining on the isotype staining. Cell suspensions were also stained for CD45, CD11b and IL-12 after LPS and IFNγ stimulation. Representative IL-12/CD11b dot plots generated from gated CD45^+^ cells are shown. The histograms below summarize the proportion of IL-12 secreting cells from tumor infiltrating CD11b^+^ cells. Purified tumor infiltrating CD11b^+^ cells were activated with LPS and IFNγ for two days and assessed for NO production (**C**). (**D**) GP33-specific T cells (as in Figure 4.A) were stimulated 24 h with GP33 in presence of CD11b^+^ cells isolated from tumors of RetCD3ε^+^ (n = 5) or RetCD3εKO (n = 5) mice. T cell inhibition by myeloid cells is determined by comparing the frequency of IFNγ secreting cells in absence and in presence of CD11b^+^ cells.

To investigate the impact of T cells on the composition of hematopoietic cells within the tumor microenvironment and in particular on tumor infiltrating myeloid cells, we crossed MT/ret mice with CD3εKO mice. We found no change in the proportion of hematopoietic cells in mice competent (RetCD3ε^+^) and deficient (RetCD3εKO) for T cells. Tumor infiltrating myeloid cells represent 79% and 81% of CD45^+^ cells (Fig. 5*A*) and express a similar level of IL-4Rα (Fig. 5*B*) in the presence or in absence of T cells respectively. Interestingly, myeloid cells derived from RetCD3εKO mouse tumors exhibit a better capacity to secreteIL-12 (Fig. 5*B*) and NO (Fig. 5*C*) than the related cells from age-matched RetCD3ε^+^ mice. Moreover, they display a poor inhibitory effect on GP33-specific T cells stimulated with GP33 compared to myeloid cells from tumors of mice competent for T cells (Fig. 5*D*). By contrast, the proportion of IL-12 producing CD11b^+^ cells in spleen and their capacity to impair T cell functions are similar in both groups of animals lpar;not shown).

### T cells favor pro-tumoral properties of tumor infiltrating myeloid cells

**Figure 6:**
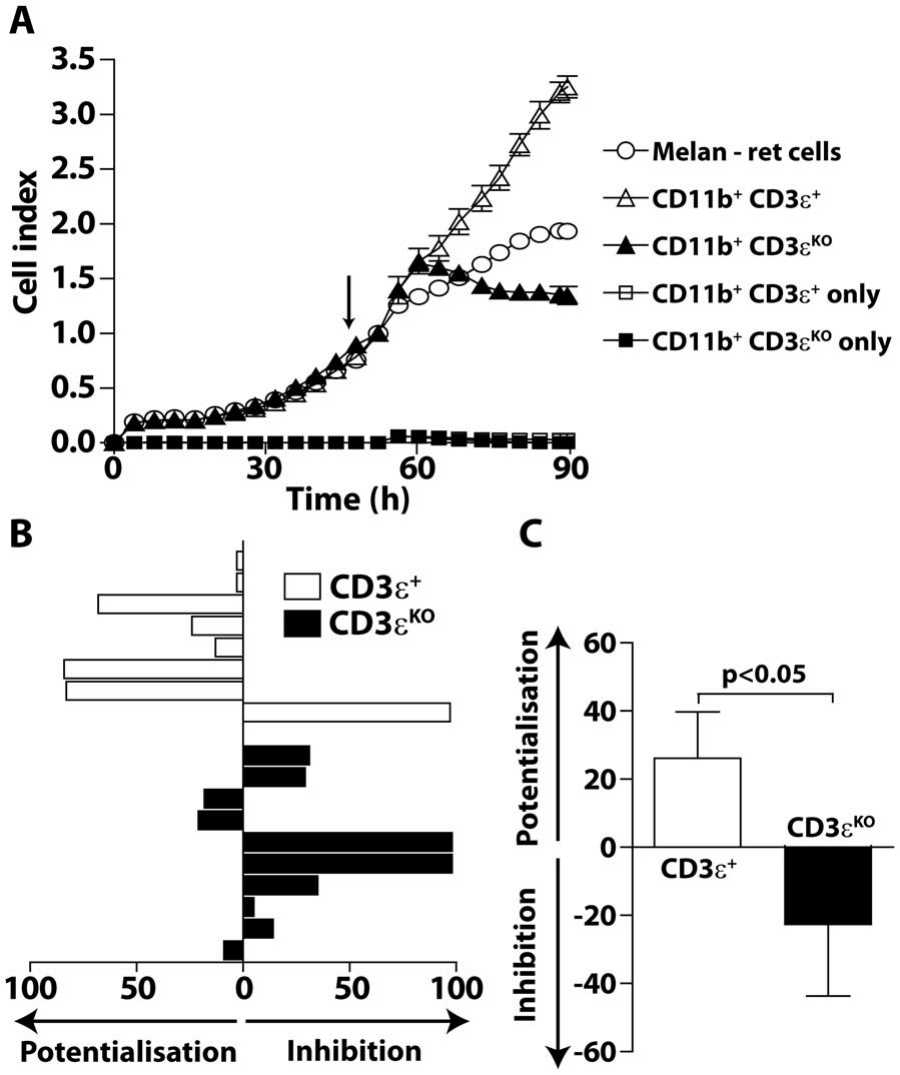
T cells contribute to the pro-tumoral properties of tumor infiltrating myeloid cells. (**A**) Melan-ret cells were seeded in the wells of E-plates. After 48 h, at the time tumor cells were in exponential growth phase (arrow), tumor infiltrating CD11b^+^ cells isolated either from RetCD3ε^+^ and RetCD3εKO mice were added and the tumor cell proliferation was assessed by dynamically monitored every 15 min on cell impedance. The graph shows the nCI values obtained by the RT-CES system for two representative CD11b^+^ cells isolated from mice deficient or competent for T cells. (**B**) The percent of inhibition of Melan-ret cell proliferation was calculated as described in Materials and Methods, 40 hours after addition of myeloid cells. (**C**) The statistical significance between the effect of CD11b^+^ cells from RetCD3ε^+^ (n = 8) and RetCD3εKO (n = 10) mice tested within three independent experiments was assessed using unpaired t test.

**Figure 7:**
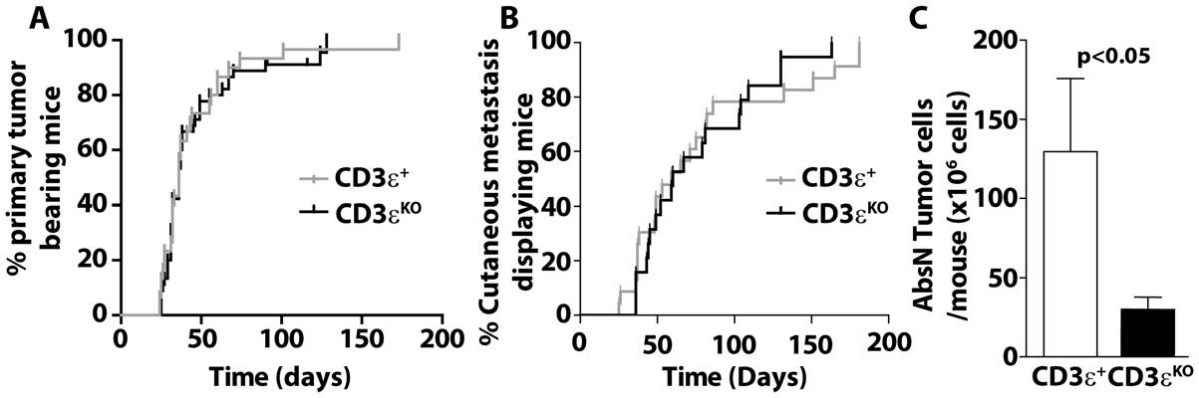
Cutaneous metastasis are smaller in T cell deficient mice than in T cell competent mice. The onset of the primary tumor (**A**) and of cutaneous metastasis (**B**) is shown for RetCD3ε^+^ (n = 16) and RetCD3εKO (n = 11) mice. (**C**) The mean absolute number of tumor cells within cutaneous metastasis per mouse is determined after enzyme digestion for more than 5 mice per group.

The proliferation of Melan-ret cells was dynamically monitored in vitro in presence of tumor derived CD11b^+^ cells from RetCD3εKO or RetCD3ε^+^ mice. These latter were added at 48 h when tumor cells reached their exponential growth phase (Fig 6A). According to their M2 phenotype, intratumoral myeloid cells from T cell competent mice promote tumor cell proliferation as shown by the cell index increase, whereas no significant cell index is recorded in wells with CD11b^+^ cells alone (Fig. 6A). Conversely, myeloid cells from RetCD3εKO mice inhibited Melan-ret cell proliferation within few hours. Indeed, in 7 cases out of 10, CD11b^+^ cells from RetCD3εKO mice inhibited from 5 to 98% of the proliferation, whereas related CD11b^+^ cells from RetCD3ε^+^ mice do it in only 1 case out of 8 (Fig 6B). After 40 hours, the tumor cell proliferation tested in three independent experiments is statistically different between the two groups (Fig 6C). Thus, our data suggest that T cells do not interfere with the recruitment of myeloid cells within the tumor microenvironment, but improve their suppressive and pro-tumoral functions. To assess whether such a different myeloid cell properties correlated with reduced melanoma progression, we diagnosed carefully melanoma symptoms in mice competent and deficient for T cells. Mice from both groups develop primary melanoma with a similar kinetic (Fig. 7*A*). No significant difference was also observed in the onset of cutaneous metastasis (Fig. 7*B*). Interestingly, at 6 month of age, the absolute number of tumor cells within metastasis per mouse is significantly lower in RetCD3εKO mice than in T cell competent mice (Fig. 7*C*), supporting a better control of metastasis growth in the absence of T cells.

## Discussion

It has been well established that myeloid cells accumulate in the spleen during tumor progression and inhibit the anti-tumor T cell response [12]. Movahedi et al identified Ly6G^-^ mononuclear and Ly6G^+^ polymorphonuclear myeloid cells in the spleen of tumor-bearing mice [3]. In MT/ret mice, splenic CD11b^+^ cells with a low or high Gr1 expression may correspond to the former and the latter fractions respectively. In the spleen of MT/ret mice, the CD11b^+^Gr1^high^ myeloid subset tends to accumulate as tumors progress, but the expansion becomes significant only at the latest stage of the disease. By contrast, myeloid cells always dominate the tumor infiltrate and the proportion of tumor infiltrating CD11b^+^ cells is associated with clinical aggressiveness. Accordingly, Soudja et al have recently shown that CD11b^+^ cells were more frequent in more aggressive melanomas in the induced melanoma model of TiRP mice [36].

We show that, during spontaneous tumor progression, M2 type marker genes (e.g. arginase1, il10, mgl1, fizz1,) are significantly upregulated in tumor infiltrating CD11b^+^ cells compared to related splenic cells. The low level of IL-4Rα on splenic myeloid cells from MT/ret mice, similar to that in negative littermates, is consistent with data in transplanted tumor models indicating that the suppressive function of splenic MDSC does not always involve a high expression of this marker [12], [37]. Arginase production by myeloid cells requires IL4 and IL13 signaling [38]. In MT/ret mice, IL-4Rα is significantly more expressed on tumor infiltrating CD11b^+^ cells than on their splenic counterparts. Accordingly, myeloid cells produce more arginase at the tumor site than in the spleen. Finally, all tumor infiltrating myeloid cells express high levels of F4/80 which clearly contrast with splenic myeloid cells that weakly express this marker. Thus, tumor microenvironment contains mononuclear cells that have differentiated into macrophages. It has been proposed that classical M1 macrophages infiltrate the site of chronic inflammation where tumors initially develop, while tumor-promoting M2 like macrophages expressing low levels of inflammatory cytokines such as IL-12 progressively replace them in established tumors [39]. Consistent with this model, the proportion of TAM able to produce IL-12 decreases in MT/ret mice with melanoma progression (not shown). Finally, spleen and tumor derived myeloid cells from MT/ret mice inhibit efficiently antigen specific T cell proliferation and IFNγ secretion. Splenic myeloid cells also impair the anti-tumor reactivity of autologous splenocytes indicating that these cells correspond to tumor-induced MDSC. Thus, tumor growth in the MT/ret model induces the replacement of normal splenic myeloid cells by suppressive cells and the recruitment of immunosuppressive myeloid cells at the tumor site.

While the role of innate immune cells in polarizing the adaptative immune response is well established [40], little is known on the reciprocal involvement of T cells in promoting the expansion and/or suppressive activity of myeloid cells [14], [31]. In a murine HPV16-induced epithelial cancer and more recently in a model of transplanted ovarian cancer, CD4^+^ T cells have been shown to promote the recruitment of myeloid cells into tumors [41], [42]. In our model, the proportion of tumor infiltrating myefloid cells is comparable in RetCD3εKO and RetCD3ε^+^ mice, indicating that their recruitment does not require T lymphocytes. Tumor infiltrating lymphocytes may rather condition intra-tumoral myeloid cells towards a M2 type macrophage profile, as suggested by data obtained in nude mice inoculated with tumor cells [43]. Our data show that tumor infiltrating myeloid cells from RetCD3εKO mice display a better capacity to secrete IL-12 and produce more NO than related cells in RetCD3ε^+^ mice. They only poorly impair the capacity of T cells to secrete IFNγ in response to antigen specific stimulation. Finally, they inhibit Melan-ret cell proliferation within few hours in contrast to intra-tumoral myeloid cells from T cell competent mice, revealing their higher cytotoxic activity towards tumor cells. Together, our data suggest that T cells affect myeloid differentiation within the tumor microenvironment and regulate their immunosuppressive and pro-tumoral properties. These data are in agreement with recent data in a model of breast cancer showing for the first time that IL4 producing CD4^+^ T cells promote pro-tumoral properties of TAM by reducing their expression level of type 1 cytokines [44].

Many reports, including ours in the MT/ret model [33], [45], support the idea that T cells exert a protective role against dissemination of metastatic cells. The present data show in addition that the genetic elimination of T cells does not accelerate the primary melanoma onset. Cutaneous metastasis develop with a similar kinetic in both RetCD3εKO and RetCD3ε^+^ mice. More surprisingly, cutaneous metastasis were smaller in T cell deficient mice than in RetCD3ε^+^ mice, suggesting a pro-tumor activity of T cells. Accordingly, DeNardo et al have recently revealed a significant role of CD4^+^ T cells as potentiators of pulmonary metastasis of mammary carcinomas through their influence on pro-tumor properties of TAM [44]. In our model, we do not privilege a pro-tumoral role of CD8 T cells, as they interfere with visceral metastasis spreading at early [33] and late [45] stages of melanoma development. Further experiments will be needed to identify which T cell subset (e.g. conventional CD4 T cells, regulatory T cells or NKT cells) was regulating intra-tumoral myeloid cell functions and what kind of mechanism is involved (e.g. IL4, IL10, IL13 or IL17) in this process in the course of melanoma progression. The unexpected clinical impact of T cell deficiency in MT/ret mice may be in part due to a direct regulation of the cytotoxic activity of myeloid cells towards tumor cells as suggested by our dynamic melanoma cell index monitoring. We cannot exclude that they also impact angiogenesis and extracellular matrix remodeling. Alternatively, the higher proportion of intra-tumoral myeloid cells able to secrete IL12 may also improve tumoricidal NK cell activity [46].

Altogether, our present data suggest that T cells establish an environment that is likely to skew tumor infiltrating myeloid cells toward a tumor promoting response. They support the existence of an immunosuppressive vicious circle in which T cells favor melanoma development by inducing a switch towards a suppressive profile of myeloid cells that, in turn, suppress T cell functions.
